# Motivated displacement assay distinguishes ALA neuron mutants from RIS neuron mutants during recovery from heat stress in *Caenorhabditis elegans*

**DOI:** 10.17912/micropub.biology.000468

**Published:** 2021-09-20

**Authors:** Carlos Chávez-Pérez, Niusha Jafari, Brendan T Keenan, David M Raizen, Alex M Rohacek

**Affiliations:** 1 Department of Neurology, Perelman School of Medicine, University of Pennsylvania; 2 Division of Sleep Medicine, Department of Medicine, Perelman School of Medicine, University of Pennsylvania

## Abstract

The interneurons ALA and RIS both regulate stress induced sleep in* C. elegans* but their roles in awake animal movement has been reported to differ. We describe the development of a motivated mobility-based assay that distinguishes between animals mutant for ALA function and those mutant for RIS function.

**Figure 1. The motivated displacement assay (MDA):**
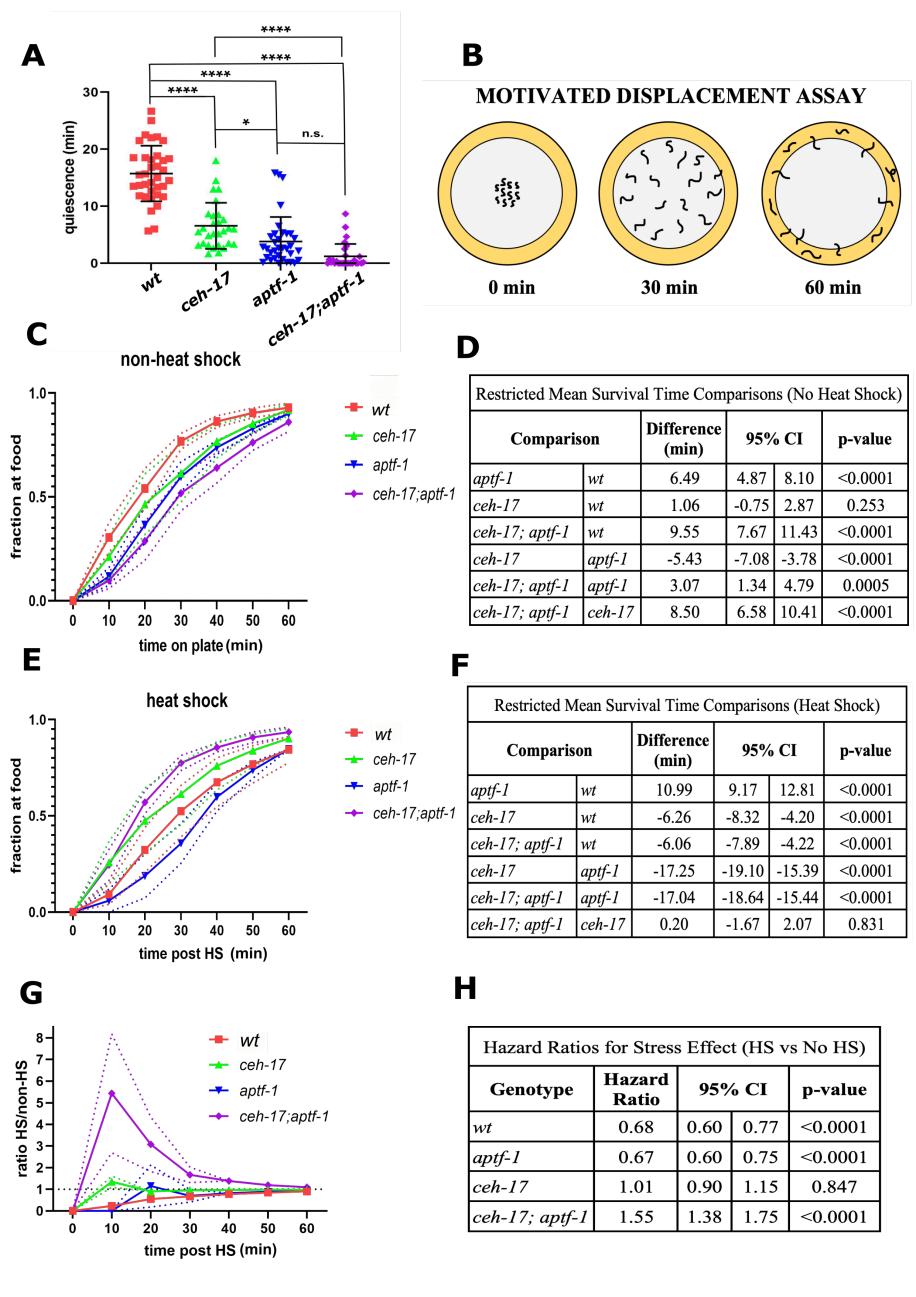
(**A**) Frame subtraction data following heat-shock of the indicated genotypes (3 replicates, with ~12 worms per replicate per genotype). ns = not significant, *P<0.05, **P<0.01, ***P<0.001, ****P< 0.0001 based on Tukey’s multiple comparison test. Horizontal bars denote means and standard deviations. (**B**) Design of the MDA. A 10 cm-diameter agar plate is seeded on its periphery with an *E. coli* 1 cm-wide ring. Worms suspended in M9 buffer are pipetted onto the center. Once the M9 dries, the worms crawl towards the food. Worms reaching the food are counted and removed from the plate every 10 minutes. Data from MDA for the indicated genotypes are shown for non-heat shocked (**C-D**) and heat shocked (**E-F**) animals, including estimates of differences in the time to reach food based on restricted mean survival time analysis. A total of 1,098 N2 (598 non-HS, 500 HS), 1,099 *ceh-17* (604 non-HS, 495 HS), 1,549 *aptf-1* (n=1,020 non-HS, 529 HS), and 1,266 *ceh-17; aptf-1* (n=585 non-HS, 681 HS) worms were studied across four trials. (**G**) Data shown in C and E is displayed as a ratio of heat-shocked to non-heat-shock worms reaching the food as a function of time. Dotted lines denote the standard error of the mean. (**H**) Hazard ratios comparing heat shocked to non-heat shock conditions within genotype are presented based on Cox Proportional Hazards model. The hazard ratio represents the relative likelihood of reaching the food in HS versus non-HS; thus, values >1 imply worms are more likely to reach food with HS than without HS, whereas values <1 imply worms are less likely to reach food with HS than without HS.

## Description

Like other animals, the nematode *C. elegans* exhibits reduced movement and sleep in response to sickness, which can be induced by exposure to high temperatures (Hill *et al.* 2014; Nelson *et al.* 2014) ultraviolet light (DeBardeleben *et al.* 2017), and other stressful exposures (Hill *et al.* 2014; Goetting *et al.* 2020). This response has been termed Stress/Sickness-Induced Sleep (SIS) (Hill *et al.* 2014; Trojanowski and Raizen 2016). Exposure to the stressor leads to quiescence in part via release of the cytokine Epidermal Growth Factor (EGF) (Hill *et al.* 2014; Konietzka *et al.* 2020), which is encoded by the gene *lin-3* (Hill and Sternberg 1992). EGF activates the ALA and RIS neurons, which then release their respective neuropeptides to effect reduced movement and behavioral quiescence (Konietzka *et al.* 2020).

Animals mutant for the homeobox gene *ceh-17* (Pujol *et al.* 2000) are defective in ALA neuron function (Van Buskirk and Sternberg 2007; Van Buskirk and Sternberg 2010). Animals mutant for the gene *aptf-1*, which encodes an interneuron-specific transcription factor (Turek *et al.* 2013), are defective in RIS neuron function. While both *ceh-17* and *aptf-1* mutants are defective in SIS (Konietzka *et al.* 2020), the two mutants are reported to behave differently. In particular, *ceh-17* mutants make dorsoventral body bends (Hill *et al.* 2014), whereas *aptf-1* mutants move only their anterior tips without significant body movement (Robinson *et al.* 2019).

Measurements of *C. elegans* sleep are commonly performed using a machine vision frame-subtraction assay, which can distinguish movement from quiescence (Raizen *et al.* 2008). However, the frame-subtraction assay does not distinguish between small ineffective movement and movement that results in displacement of the animal. Indeed, when assessed using this frame-subtraction method, both *ceh-17* and *aptf-1* single mutants, as well as *ceh-17; aptf-1* double mutants, show reduced quiescence following a heat stressor (**Fig. 1A**). Hence, the frame-subtraction machine vision assay does not distinguish *ceh-17* from *aptf-1* mutants.

In order to distinguish these two mutants, we designed a motivational displacement assay (MDA) (**Fig. 1B**). The MDA, which has also been termed the “Edge Assay” (Kawamura and Maruyama 2019), is a modification of the “Food Race Assay” (Mitchell *et al.* 2010), only instead of providing animals with a point source of motivating food, we provide them with a ring of food. Worms are placed in the center of a 10cm diameter circular petri dish with NGM agar and a ring of bacterial food at the dish periphery acting as the motivation source. Worms that reach the food are counted and removed from the assay plate at 10-minute intervals. The MDA can be used to compare genotypes under unstressed conditions, as well as to assess effects of stress (HS) on motivated movement. We performed this assay for *ceh-17* and *aptf-1* single mutants as well as for *ceh-17; aptf-1* double mutants under non-heat shock (non-HS) and under 30 minute 35°C heat shock (HS) conditions. We made a number of observations.

First, under non-HS conditions, we observed a significant difference among genotypes in time to reach the food (p <0.0001). There was no significant difference between *ceh-17* and *wild type* animals in the time to arrive at the food (mean difference [95% Confidence Interval] = 1.06 [-0.75, 2.87] minutes; p=0.253), but both *aptf-1* single mutants (6.49 [4.87, 8.10] minutes slower; p<0.0001) and *ceh-17; aptf-1* double mutants (9.55 [7.67, 11.43] minutes slower; p<0.0001) reached the food significantly slower than *wild type* worms (**Fig. 1C-D**). This observation indicates that *aptf-1* is required for movement to food even in the absence of stress. Three possible explanations, which are not mutually exclusive, include (1) reduced ability of *aptf-*1 mutants to sense the food, (2) reduced motivation of *aptf-*1 mutants the find the food, and (3) reduced ability of *aptf-*1 mutants to move effectively. While we cannot yet distinguish between these explanations, we favor the third based on prior observations of the movements made by *aptf-1* mutants (Robinson *et al.* 2019).

Second, under HS conditions (**Fig. 1E-F**), we again found a significant overall difference among genotypes (p <0.0001). In subsequent pairwise comparisons, we observed that *aptf-1* mutants reached the food significantly slower than wild-type worms (10.99 [9.17, 12.81] minutes slower; p<0.0001), while *ceh-17* worms reached the food faster than *wild type* (6.26 [4.20, 8.32] minutes faster; p<0.0001). Interestingly, under HS conditions, the *ceh-17; aptf-1* double mutants reached the food significantly faster than *wild type* (6.06 [4.22, 7.89] minutes faster; p<0.0001) and at virtually the same speed as *ceh-17* single mutants (p=0.831).

Lastly, we compared the effects of heat shock within and between genotypes using a Cox proportional hazards (PH) model. The results of a statistical interaction test between stress and genotype showed that the response to heat shock differs across genotypes (p<0.0001; **Fig. 1G**–**H**). To better understand these effects, we conducted stratified analyses based on genotype in the context of the Cox PH model. *ceh-17* mutants showed no response to heat shock (HR [95% CI] = 1.01 [0.89, 1.14]; p=0.847), whereas *aptf-1* (0.67 [0.60, 0.75]; p<0.0001) and *wild type* worms (0.68 [0.60, 0.77]; p<0.0001) were less likely to reach the food after heat shock than without heat shock. Finally, *ceh-17; aptf-1* double mutants were more likely to reach the food after heat shock than without heat shock (1.55 [1.38, 1.75]; p <0.0001).

In conclusion, the MDA can distinguish between mutants that affect the ALA neuron and mutants that affect the RIS neuron. The MDA also acts as an additional tool to assess the behavior before and after heat shock. In particular, our observations from the MDA suggest that *ceh-17; aptf-1* double mutants have a stronger movement deficiency than *aptf-1* single mutants in the no HS condition, but reach the food similar to *ceh-17* single mutants under HS conditions. One possible explanation for these observations is that the ALA and RIS circuitry is altered by heat shock. Further study into the circuitry of the ALA and RIS neurons is warranted.

This assay can be helpful in the characterizations of newly identified SIS mutants, and for enriching for SIS mutants when performing forward genetic screens. Our preliminary observations suggest that the MDA can also be used to assess effects of other stressors.

## Methods

**MDA assay plate preparation**

To prepare assay plates, we poured 35.0 mL of molten NGM agar into 10.0 cm-diameter plates and let the agar solidify at room temperature for two days. DA837 *E. coli* (Davis *et al.* 1995) grown in liquid LB media was pipetted along the periphery of the plates creating a ring with a width of approximately one centimeter. We placed the seeded plates in a 37°C incubator to grow the bacteria overnight. We used the alkaline bleach method (Stiernagle 2006) to obtain synchronized populations of first larval stage (L1) worms. We pipetted approximately 150 synchronized L1’s suspended in M9 buffer onto a six-cm diameter NGM plates pre-seeded with *E. coli* bacteria, which constitutes the worm’s diet. The worms were cultivated in a 20°C incubator for three or four days until day 1 or day 2 of adulthood, respectively. The animals are then tested during their first or second day of adulthood.

On the day of the assay, adult worms of each genotype were washed off the six-cm plates with 7mL of M9 solution and transferred into a 15mL conical tube. They were centrifuged in a table-top clinical centrifuge for 30 seconds at maximum speed. The supernatant was removed, and the worm pellet was washed with 7mL of M9 to remove remaining bacteria before being centrifuged again for 30 seconds at maximum speed. The supernatant was removed leaving the worms suspended in approximately 100uL of M9 in the conical tube. We pipetted 35uL of the resuspended worms (~150 worms) onto the agar surface in the geometrical center of 10 cm-diameter round assay plates that had been warmed to 35°C overnight. After adding the worms, the plates were sealed with two layers of Parafilm and placed in a 35°C incubator for 30 minutes. A 35°C exposure constitutes a heat shock to the worms, which prefer temperatures between 15-25°C. For the control (non-heat shock), peripherally seeded 10cm-diameter plates were left at room temperature (22-23°C) overnight. Worms were prepared as above and 35uL of worm solution containing approximately 150 adult worms was pipetted onto the agar surface at the geometrical center of an assay plate and the droplet was allowed to absorb into the agar. For the non-HS condition, time 0 was defined as the time at which the droplet had been completely absorbed, allowing for worms to begin migrating away from the plate center. For the heat shocked plates, time 0 was immediately after the 30 min heat shock (the pellet was determined to have absorbed in this time and no worms had migrated to the food). The plates were observed every 10 minutes for one hour. Animals that had reached the food ring were recorded and removed using a Pasteur pipette connected to vacuum suction. At the end of the one-hour period, we counted the total number of worms remaining on the plate.

**Statistical analysis**

Statistical analyses focused on understanding differences between genotypes, including comparisons of overall quiescence and time to reach food during non-heat shock (unstressed) and heat shock (stressed) conditions. Comparisons of quiescence among genotypes were performed using an analysis of variance (ANOVA), followed by pairwise comparisons using Tukey’s multiple comparisons test. To evaluate differences in time to reach food, we utilized complementary survival analysis approaches including Cox Proportional Hazards (PH) models and Restricted Mean Survival Time (RMST) analysis. Cox PH models were used to evaluate the effect of heat shock within each genotype and test whether these effects differed by genotype, while controlling for the effect of any differences across the four experiments. Specifically, differences in the response to heat shock by genotype were assessed using a statistical interaction test evaluating the significance of the product term (heat shock [Yes, No] × genotype [*wild type, aptf-1, ceh-17, ceh-17; aptf-*1]) in a model that also included both main effect terms (heat shock and genotype) and a covariate for replicate. Assessments of the response to heat shock stratified by genotype and differences among genotypes stratified by heat shock exposure were similarly performed in the context of Cox PH models controlling for experiment. Results from Cox PH models are presented as hazards ratios (HRs) and 95% confidence intervals (CIs), representing the difference in the likelihood of reaching the food between genotypes or during heat shock vs. no heat shock. While graphical analysis supported the proportional hazards assumption of the Cox PH model, to evaluate any potential impact of deviation from this assumption on observed results we also performed pairwise comparisons of stratums using RMST analysis. Results of RMST analyses are reported as the differences in mean survival time and associated 95% CIs between groups. The findings from RMST analysis were consistent with the findings from Cox PH models. A significance level of α = 0.05 were used for all tests. All analysis were performed in SAS software, version 9.4 (SAS Institute Inc., Cary, NS) and STATA software, Version 14 or 16 (StataCorp, College Station, TX).

## Reagents

**Reagents**

**Table:** 

**Strain**	**Genotype**	**Available from**	**Reference**
N2	*wild type*	CGC	(Brenner 1974)
IB16	*ceh-17 (np1) I*	CGC	(Hill *et al.* 2014)
HBR232	*aptf-1 (tm3287) II*	CGC	(Turek *et al.* 2013)
NQ1065	*ceh-17(np1) I; aptf-1 (tm3287) II*	Raizen Lab	(Grubbs *et al.* 2020)
DA837	Streptomycin-Resistant OP50	CGC	(Davis *et al.* 1995)
